# Costello Syndrome with Severe Nodulocystic Acne: Unexpected Significant Improvement of Acanthosis Nigricans after Oral Isotretinoin Treatment

**DOI:** 10.1155/2015/934865

**Published:** 2015-02-28

**Authors:** Leelawadee Sriboonnark, Harleen Arora, Leyre Falto-Aizpurua, Sonal Choudhary, Elizabeth Alvarez Connelly

**Affiliations:** ^1^Division of Dermatology, Department of Pediatrics, Faculty of Medicine, Khon Kaen University, Khon Kaen 40002, Thailand; ^2^Miller School of Medicine, Department of Pediatric Dermatology, University of Miami, 1600 NW 10th Avenue, Rosenstiel Medical Science Building, Room 2023, Miami, FL 33136, USA

## Abstract

We report the case of 17-year-old female diagnosed with Costello syndrome. Genetic testing provided a proof with G12S mutation in the HRAS gene since 3 years of age with a presentation of severe nodulocystic acne on her face. After 2 months of oral isotretinoin treatment, improvement in her acne was observed. Interestingly, an unexpected significant improvement of acanthosis nigricans on her neck and dorsum of her hands was found as well. We present this case as a successful treatment option by using oral isotretinoin for the treatment of acanthosis nigricans in Costello syndrome patients.

## 1. Introduction

Costello syndrome is an autosomal dominant inherited disorder with frequent de novo mutation in the HRAS gene [1]. This syndrome has no formal diagnostic criteria. However, it has characteristic craniofacial appearances, hand and wrist posture abnormalities, musculoskeletal and cardiac abnormalities, neurologic problems, psychomotor developmental delay, intellectual disability, and failure to thrive. Cutaneous manifestations in Costello syndrome are common and were described in many literatures [2–4] which included papillomatosis, palmoplantar keratoderma, follicular hyperkeratosis, and acanthosis nigricans [3]. According to up-to-date knowledge, there was no favorable treatment option for these cutaneous findings.

## 2. Case Presentation

**Figure 1:**
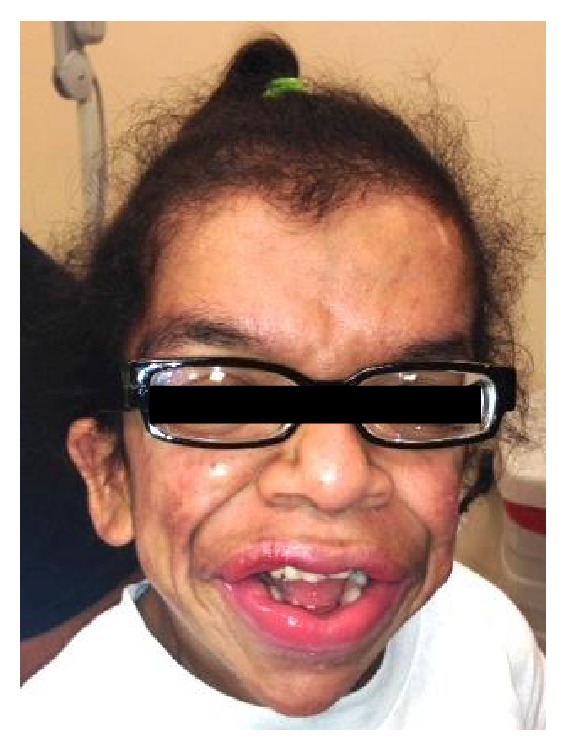
Facial manifestation in Costello syndrome.

A 17-year-old female was first seen in January 2013 for her complaining of multiple nodulocystic acne on the face persisting for several months. Diagnosis with Costello syndrome was proved since the age of 3 by genetic testing which revealed a mutation (G12S) in the HRAS gene. She also has typical features of this syndrome which included coarse facies (Figure 1), sparse hair, full lips, redundant skin, ulnar deviation of the wrists and fingers, developmental delay, and hydrocephalus which has been operated and drained for several occasions by pediatric neurologist. Hyperkeratotic hyperpigmented plaques on dorsal aspect of her hands and acanthosis nigricans around her neck were also seen and described as typical skin findings commonly found in Costello syndrome as well.

**Figure 2:**
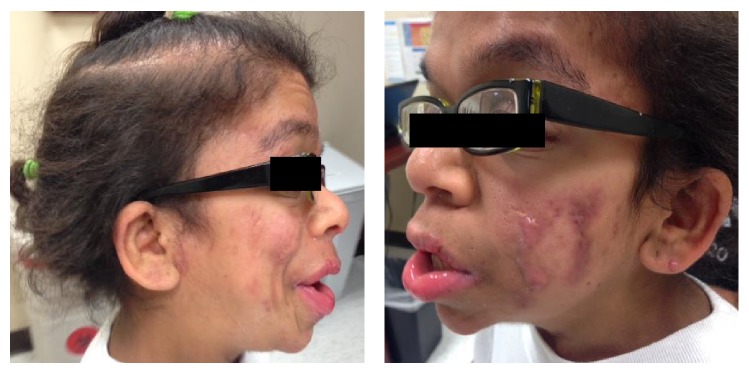
Improvement of nodulocystic acne on the face after 2 months of oral isotretinoin treatment.

**Figure 3:**
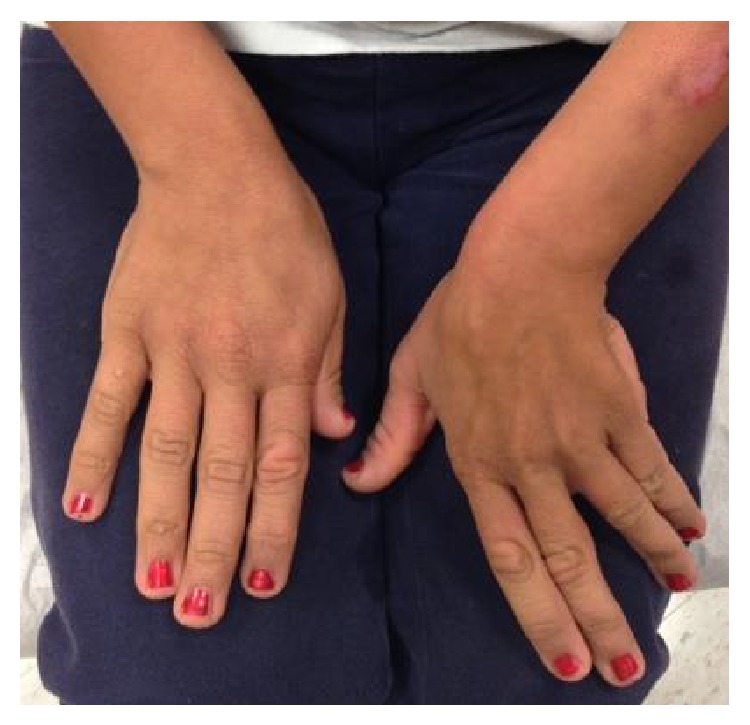
Significant improvement of acanthosis nigricans and hyperkeratotic hyperpigmented plaques on the hands after 2 months of oral isotretinoin.

According to her severe nodulocystic acne manifestation, literatures were reviewed and, to date, there was no association between this syndrome and severe nodulocystic acne. However, a prominent history of severe acne with current residual scarring in both her parents was the evidence supporting that severe acne manifestation runs in her family. We then decided to treat her acne initially with oral tetracycline and short course prednisolone 0.5 mg/kg/day without improvement. During that time blood tests were performed including (1) complete hormone panels to exclude adrenal source of hormones since the patient has never menstruated and (2) preisotretinoin laboratory evaluations included the pregnancy testing. All results were within normal limits and she was then approved to register in the iPledge system. After discussing risks and benefits, isotretinoin was started at 20 mg orally once a day with significant improvement of her acne after 2 months of the drug administration (Figure 2). Without anticipation, the hyperkeratotic hyperpigmented plaques on dorsal aspect of her hands and the acanthosis nigricans around her neck also had significant improvement from the same isotretinoin treatment as well (Figure 3).

## 3. Discussion

Costello syndrome is an autosomal dominant inherited disorder with frequent de novo mutation, initially described by Costello [5] in 1971 and 1977. In 2006, Kerr et al. [6] found a heterozygous missense mutation in the protooncogene HRAS where, to date, it has been well accepted that Costello syndrome is caused by HRAS mutations only. Diagnosis of Costello syndrome can be made by typical characteristic of the craniofacial appearances and musculoskeletal, cardiovascular, and neurologic abnormalities as well as psychomotor developmental delayed as described as follows.

*List of Abnormalities Found in Costello Syndrome [1]*

*Craniofacial Appearance*

- Coarse facial features; full cheeks, full lips, large mouth, and full nasal tip,
- curly, sparse, fine hair,
- wide nasal bridge.

*Musculoskeletal System*

- Diffuse hypotonia and joint laxity,
- ulnar deviation of wrists and fingers and splayed fingers,
- spatulate finger pads and abnormal finger nails,
- positional foot deformity.

*Cardiovascular System*

- Cardiac hypertrophy,
- congenital heart defects,
- aortic dilatation.

*Neurologic Abnormalities*

- Hydrocephalus,
- tethered cord,
- Chiari I malformation.

*Psychomotor Development*

- Developmental delay,
- intellectual disability.

Cutaneous manifestations in Costello syndrome are common and were described in many literatures. Nguyen et al. [3] collected the reported cutaneous signs of Costello syndrome which included (1) loose/redundant/lax skin, (2) deep palmar and/or plantar creases, (3) sparse or thin, curly hair, (4) hyperkeratosis, hyperpigmentation (generalized, localized, or acanthosis nigricans), (5) dysplastic/thin/brittle or deep-set nails, and (6) papillomas.

In our case, the patient showed the nucleotide substitution c.34G > A, resulting in p.Gly12Ser amino acid change representing a mutation (G12S) in the HRAS gene. The patient is also presented with the typical facial manifestations, hand and wrist posture abnormalities, hydrocephalus, and marked cutaneous findings of hyperkeratosis and acanthosis nigricans. Another cutaneous finding found in our patient was the manifestation of severe nodulocystic acne where, to date, literatures reviewed have no association between this finding and Costello syndrome. However, a prominent history of severe acne with current residual scarring in both her parents was the evidence supporting that severe acne manifestation runs in her family.

To date, acanthosis nigricans is apparently a reaction to many stimuli. Many disorders associated with this skin finding included obesity, endocrinologic disorders, and malignancy. Treatment of the underlying disorders which triggered this skin manifestation is the treatment of choice. However, it is possible that acanthosis nigricans which presented in our patient may serve as a direct correlation with HRAS mutation associated with Costello syndrome. Moreover, we have explored other possible related disorders. There were no significant abnormal lipid profiles found from her blood test. Her body weight was normal. The endocrinologic disorders were screened according to the delay of her menstrual period and the results were all normal.

In general, the treatment of hyperkeratosis and acanthosis nigricans in Costello syndrome is mainly for cosmetic purpose; therefore, there was no previous treatment for the cutaneous findings in this patient. Other treatments that have been used in the treatment of acanthosis nigricans are topical keratolytic agents such as urea, salicylic acid, topical tretinoin [7], hydroquinone, and ammonium lactate with variable results. Systemic agents which showed benefits in some case reports are etretinate [8], isotretinoin, and metformin [9]. Resolving of acanthosis nigricans around her neck and decreasing of hyperkeratotic hyperpigmented plaques on dorsum of her hands in our patient were interestingly found after 2-month course of oral isotretinoin for the treatment of her acne. We report this finding as a successful treatment option for acanthosis nigricans and hyperpigmented skin found in Costello syndrome.
